# Editorial: Precision therapeutics in rare cancers: targeting tumor microenvironment and biomarker-driven approaches

**DOI:** 10.3389/fimmu.2026.1884890

**Published:** 2026-05-29

**Authors:** Takahiro Tsujikawa, Elise F Nassif Haddad, Emanuela Palmerini, Ramon W. Mohanlal

**Affiliations:** 1 Department of Otolaryngology–Head and Neck Surgery, Kyoto Prefectural University of Medicine, Kyoto, Japan; 2 Department of Sarcoma Medical Oncology, MD Anderson Cancer Center, Houston, TX, United States; 3 Sylvester Comprehensive Cancer Center, Miller School of Medicine, University of Miami, Miami, FL, United States; 4 RND Pharmaceuticals, Inc., New York, NY, United States

**Keywords:** biomarker, precision medicine, rare cancer, rare tumor, tumor micro environment (TME)

Rare tumors, such as sarcoma and glioblastoma, have an incidence of fewer than six cases per 100,000 individuals annually; however, taken together, they account for up to 24% of all human cancers and 30% of cancer-related deaths, making them one of the significant challenges for cancer care, research, and therapeutic development (1). Rare tumors represent a uniquely challenging domain within oncology due to substantial etiological heterogeneity and the limited availability of large−scale clinical datasets. This often impedes the development of evidence−based therapeutic strategies and slows the translation of emerging discoveries into clinical practice. At the same time, advances in our understanding of immunotherapy, multi−omics profiling, and the tumor microenvironment (TME) may enable the application of precision medicine approaches even in small patient populations. Rare tumors frequently harbor distinct molecular features and immune signatures, making them particularly suitable for biomarker−guided approaches and innovative therapeutic designs. This Research Topic aimed to highlight recent progress in TME−focused biology, biomarker discovery, and precision therapeutics across a spectrum of rare tumors. Through eight manuscripts, this Research Topic provides insights into immunosuppressive mechanisms, prognostic modeling, molecular classification, and determinants of immunotherapy response.

To explore mechanisms of tumor−driven immune suppression, Wang J. et al. reviewed the emerging role of SPP1^+^ macrophages in a broad range of tumors, including rare tumors, detailing how this population contributes to immunosuppressive TMEs and discussing therapeutic strategies targeting these cells. SPP1^+^ macrophages are a subset of tumor-associated macrophages (TAMs) that contribute to cancer progression through multiple TME-related mechanisms. In the field of biomarker−driven modeling, Wang Y. et al. constructed a prognostic signature based on palmitoylation−related lncRNAs, offering a framework for predicting outcomes and drug benefit in breast cancer. Cao et al. provided a comprehensive review of PD−L1 biology in Primary Central Nervous System Diffuse Large B−cell Lymphoma (PCNS−DLBCL), showing that in some patients, the TME exhibited high levels of PD-L1, which may be linked to prognosis. PD-L1 expression was associated with the amplification of the 9p24.1 copy number in lymphoma cancer cells, acting as a key immune evasion mechanism. This article summarized molecular mechanisms and evaluated the translational potential of PD−L1−targeted therapies. Shen et al. examined NK−cell heterogeneity, highlighting how NK−cell diversity in physiological and tumor contexts may inform future immunotherapeutic strategies. Single-cell sequencing technology revealed a high degree of heterogeneity in gene expression profiles between individual NK cells, which may be used as the basis of personalized treatment strategies with NK cells.

Several studies in this Research Topic focused on the immune microenvironment of rare tumors, as shown in the following contributions. First, Zeng et al. characterized distinct genomic and immune features in *KRAS*−mutated mesonephric−like adenocarcinoma (MLA) subtypes, revealing biologically meaningful differences between *KRAS*^mut^/*SPOP*^mut^ and *KRAS*^mut^/*PIK3CA*^mut^ tumors, which may have therapeutic implications. In particular, expression levels of the cytokines cIFNG, IFN6, and IFN1 in the *KRAS*^mut^/*SPOP*^mut^ group were significantly lower than the levels found in the *KRAS*^mut^/*PIK3CA*^mut^ group. This is associated with reduced immune cell infiltration in the TME.

Silva et al. profiled pediatric germ cell tumors (GCTs) and identified key immune cell populations and potential therapeutic targets, contributing valuable insights into this rare pediatric malignancy. Dysgerminomas exhibited an immune-active profile with elevated levels of T cells, CD8^+^ T cells, and cytotoxic cells, suggesting potential responsiveness to checkpoint inhibitors. In contrast, yolk sac tumors displayed an immunosuppressive environment with high CD24 and PVR expression, indicative of unique immune evasion mechanisms. Embryonal carcinomas also showed high CD24 expression. This study provides new insights into pediatric GCT immunology, supporting the potential of tailored immunotherapeutic strategies targeting the distinct immune profiles of pediatric GCT histologies.

Jiang et al. presented a case report describing long−term survival in anorectal malignant melanoma treated with cryoablation and adjuvant PD-1 inhibitor therapy with Toripalimab, illustrating the potential of combining local and systemic immunotherapies in treating rare aggressive tumors.

Li et al. analyzed dynamic changes in peripheral blood lymphocyte subsets in patients with metastatic osteosarcoma receiving immune checkpoint inhibitors, demonstrating that early shifts in T−cell and NK−cell populations were predictive of treatment efficacy and prognosis.

Finally, Tsujikawa et al. investigated the clinical, genomic, and immune determinants of nivolumab response in head and neck squamous cell carcinoma, identifying features associated with therapeutic sensitivity and resistance. Biomarker profiles for peripheral NLR, T-cell densities, and lymphoid-inflamed profiles, along with tumor suppressor gene pathways (*TP53*, *CDKN2A*, and *SMAD4*), were correlated with response versus non-response to the PD1-inhibitor nivolumab.

Taken together, these studies underscore the importance of integrating TME biology, molecular profiling, and immunologic insights to advance precision therapeutics in rare cancers. Looking ahead, several areas warrant continued attention. First, a deeper characterization of the TME, including macrophage subsets, NK−cell states, and stromal interactions, will be essential for designing next−generation immunotherapies. Second, harmonized frameworks for biomarker validation and predictive test development will be critical for translating molecular discoveries into actionable clinical tools. Finally, collaboration between the pharmaceutical industry and the medical/scientific community, global collaboration in general, and data sharing will play a central role in accelerating progress by enabling researchers to pool resources, validate findings, and expand therapeutic opportunities for patients with rare cancers.

Overall, the articles in this Research Topic review highlight the importance of taking a personalized approach to therapy development for rare tumors targeting the TME, which will require the development of predictive tests to enable the use of these precision therapeutics.
